# Symptoms of Mood Disturbance and Depression Diagnosis Among South Asian Home Care Clients in Ontario, Canada: Evidence of Under‐Detection of Mental Health Needs

**DOI:** 10.1002/gps.70201

**Published:** 2026-03-17

**Authors:** Priyamadhaba Behera, Navjot Gill, Heebah Sultan, George A. W. Heckman, John P. Hirdes

**Affiliations:** ^1^ Department of Community Medicine and Family Medicine All India Institute of Medical Sciences Bhubaneswar India; ^2^ Research Institute for Aging and School of Public Health Sciences University of Waterloo Waterloo Ontario Canada; ^3^ Ontario Health Toronto Ontario Canada; ^4^ St. Joseph's Health Centre Parkwood Hospital London Ontario Canada; ^5^ School of Public Health Sciences University of Waterloo Waterloo Ontario Canada

**Keywords:** depression, interRAI home care, mood disturbance symptoms, South Asia

## Abstract

**Background:**

Depression poses a significant global health burden yet remains widely undiagnosed and untreated, especially among South Asian populations. Despite higher prevalence rates, depression among South Asian immigrants in countries like Canada is often under‐recognized due to cultural nuances. This study aims to examine patterns of mood disturbance and risk factors for depression diagnosis among South Asian and general home care clients in Ontario, Canada.

**Methods:**

Using data from the interRAI Home Care (HC) assessments conducted between 2018 and 2022, demographic and clinical characteristics of South Asian home care clients were compared against the general home care population. Depression diagnosis and mood disturbance severity were assessed using standardized measures. Multivariate logistic regression models were employed to examine risk factors for depression diagnosis.

**Results:**

While mood disturbance prevalence was comparable, the percentages with depression diagnosis were significantly lower 0.50 (95% CI 0.47–0.54) among South Asian home care clients compared to the general home care population. Multivariate analyses confirmed this difference even after accounting for demographic and clinical factors.

**Discussion:**

The under‐detection of depression among South Asian home care clients suggests potential issues related to cultural competence among health care providers and stigma. Systematic assessment tools like the interRAI HC can aid in identifying mental health needs. Efforts are needed to increase awareness, reduce stigma, and provide culturally appropriate mental health services for South Asian populations.

**Conclusion:**

Depression is under‐recognized among South Asian home care clients in Ontario, Canada, despite a similar prevalence of mood disturbance. Addressing cultural competence and stigma is crucial for improving the detection and treatment of depression in this population.

## Key Points

- South‐Asian home care clients have similar levels of mood disturbance to the general home care population, but depression appears to be under‐diagnosed
- Under‐diagnosis of depression in South‐Asian home care clients persists after adjusting for demographic characteristics, clinical indicators, protective variables, and risk factors
- Cultural consideration such as stigma, atypical symptom presentation, and cultural competence of clinicians may contribute to under‐detection
- Standard assessments like the interRAI Home Care can support improved detection and response to mood disturbance in cultural minorities

## 1 Introduction

Depression is a significant mental health disorder affecting an estimated 280 million people worldwide [1]. Despite this substantial burden, many individuals with depression, particularly in low‐ and middle‐income countries (LMICs), do not receive adequate care [2]. The global treatment gap for depression exceeds 75% in many LMICs, indicating that three out of four people with depression go untreated [3]. Depression is the most common geriatric psychiatric disorder and a major risk factor for disability and mortality in older patients [4, 5]. A systematic review and meta‐analysis by Zenebe et al. [6] estimated the pooled prevalence of depression among older adults to be 31.74% (95% CI: 27.90%–35.59%), underscoring a significant mental health burden among the older population.

In South Asia, including countries such as India, Pakistan, Bangladesh, and Nepal, depression is deeply influenced by diverse cultural norms, family structures, and religious beliefs [7]. These sociocultural factors significantly shape how depressive symptoms are experienced, expressed, and interpreted [7, 8]. Rather than articulating emotional distress, individuals often present with somatic symptoms such as fatigue, sleep disturbances, or pain, which can lead to underdiagnosis in clinical settings not attuned to these cultural patterns [7, 9, 10]. Close‐knit family systems and religious beliefs may offer protective support, yet they can also reinforce stigma, guilt, and a reluctance to seek professional help [11]. Further, globalization and rapid social change may exacerbate mental health stressors while traditional models of care remain limited [12]. These cultural dynamics may complicate both the accurate estimation of depression prevalence and its clinical recognition in South Asian populations in Canada.

Among South Asian immigrant populations in Western countries such as Canada and the United Kingdom, evidence suggests a disproportionately high burden of depression [13, 14]. Studies have reported elevated depressive symptoms among South Asian women in the UK (approximately 30%) and a 2.5‐fold higher risk of depression among South Asian immigrants in Canada compared to non‐immigrants [15, 16]. However, despite this elevated need, mental health service utilization remains low [14]. Specifically, South Asian individuals in Canada experiencing a major depressive episode reported the highest proportion of unmet mental health care needs (48%) and the greatest perceived barriers to accessing care (33%) compared to other ethnic groups [17]. This underutilization has been attributed to multiple barriers, including language difficulties, stigma, limited awareness of available services, and a lack of culturally sensitive care models [18].

In Canada, home care plays a vital role in supporting the health and well‐being of older adults by providing services that promote independence, manage physical health needs, and improve quality of life [18]. However, formal home care services are underutilized among South Asian (SA) immigrant communities due to culturally rooted familial expectations, where caregiving is viewed as a moral obligation and a source of family honor [19, 20]. Multigenerational households commonly assume caregiving responsibilities, with adult children often acting as primary caregivers, reflecting strong cultural values of filial piety and family cohesion [21, 22]. While these family‐based arrangements promote resilience, they may also contribute to caregiver burden, unmet healthcare needs, and limited engagement with formal health services, including mental health care [18, 22]. Evidence indicates that depression among SA immigrants is frequently underdiagnosed and undertreated, partly due to the atypical somatic presentation of depressive symptoms and stigma around mental illness within this community [10, 23]. This results in a significant treatment gap, where despite high prevalence rates, many individuals do not receive adequate mental health care [18, 22].

Despite the growing population of aging SA immigrants in Canada, evidence about the mental health needs of home care clients is very limited [24, 25]. The result is that policy makers have inadequate information about the magnitude of the problem, clinicians and service providers may fail to recognize and respond to mental health needs, and harm may be experiences by older SA home care clients not receiving adequate mental health care. This study aims to fill a critical knowledge gap by examining symptoms of mood disturbance and depression diagnosis among South Asian home care clients in Ontario, Canada.

## 2 Methods

### 2.1 Study Design

We completed secondary analyses of clinical assessment and administrative records in a cross‐sectional observational study of a population of persons receiving long‐stay home care services.

### 2.2 Setting, Participants, and Inclusion Criteria

Adult long‐stay home care recipients (*n* = 428,373) in Ontario, Canada expected to be on service for 60 days or longer were studied to examine patterns of mood disturbance and risk factors for a depression diagnosis among South Asian clients and the general home care population. All adult long‐stay home care recipients assessed with the interRAI Home Care [26] as part of routine clinical practice between 1^st^ April 2018 and 31^st^ December 2022 were included in this study. Even though the interRAI HC is used in 10 provinces/territories in Canada, the study was restricted to Ontario because that was the only province in which we had access to language codes and there were enough cases to minimize reidentification risk. Although the sample includes home care clients from all regions of Ontario, including remote rural areas, it should be noted that the South Asian population of Ontario is largely concentrated in major urban areas, particularly in cities near Toronto.

### 2.3 Dependent Variables

The two main dependent variables of interest were the presence of a depression diagnosis and symptoms of mood disturbance. Care coordinators use existing clinical records (e.g., hospital discharge summaries, primary care referrals, agency health records) as well as direct interviews with clients and their caregivers to determine whether the client has been formally diagnosed by a health professional. The Composite Mood Scale (CMS) was used to measure symptom severity of mood disturbance at the time of assessment [26]. The CMS was developed using interRAI assessments from nine health sectors, including mental health services, long‐term care, primary care, hospitals, palliative care, and general population samples. It has been validated against diagnosis and indicators of self‐harm, and the self‐reported version of this scale was validated against the Kessler‐10 measure of psychological distress, demonstrating strong convergent validity (*r* = 0.73). The CMS has been more sensitive to current mood disturbance than reliance on diagnosis or the Depression Rating Scale alone, particularly in populations where depression may be underdiagnosed [26]. The CMS includes items related to dysphoria, anxiety, and anhedonia based on both the person's self‐reported mood and clinical ratings to provide a combined measure of severity of mood disturbance with scores ranging from 0 (no indicators present) to 9 (severe mood disturbance) [26].

### 2.4 Independent Variables

The home care assessment includes various care protocols, summary scales, and decision‐support algorithms to support care planning and determine the nature and intensity of the person's needs. The main clinical summary scales we used as independent variables included the Cognitive Performance Scale for cognitive function with scores ranging from 0 (independent) to 6 (severe cognitive impairment) [27]; the Activities of Daily Living (ADL) Hierarchy Scale for functional impairment with scores ranging from 0 (independent) to 6 (total dependence) [28]; and the Changes in Health, End‐stage Disease, and Signs and Symptoms (CHESS) scale for health instability with scores ranging from 0 (no instability) to 5 (severe instability in health) [29]. Aside from demographic variables (e.g., age, gender, marital status), we examined other risk factors likely to be associated with depressive symptoms such as major life stressors, strong and supportive family members, and economic trade‐offs. In addition, we considered indicators that might considered to be indicators for depression that are not included in the CMS (e.g., pain, sleep disturbance, fatigue, infrequent meals).

### 2.5 Data Sources and Measurement

Ontario has implemented a stepped approach to home care assessments, which includes using the interRAI Contact Assessment (CA) at intake and the interRAI Home Care (HC) assessments for long‐stay home care clients [30]. The interRAI HC is a standardized, comprehensive, and internationally validated clinical assessment comprising of approximately 250 items [31, 32]. Care coordinators use all sources of information to complete the assessment, including direct interviews and observations of clients, discussions with family and caregivers, reviews of available charts and other relevant documentation, and consultations with other health professionals. They exercise their clinical judgment to determine the most appropriate response based on this information using standardized definitions and coding guidelines for each item in the assessment. The interRAI HC is mandated in Ontario to be the standard assessment for all clients expected to receive home care services for 60 days or more. It should be noted that the interRAI HC is intended to be a comprehensive assessment of functional status, symptom severity, social resources, and service allocations; however, it is not intended to be a diagnostic tool.

As part of routine practice, care coordinators in home care ascertain and record the client's primary language at intake. South Asian clients were identified using these data on their primary language matched to International Organization for Standardization (ISO) language codes. Members of the research team with South Asian backgrounds collaborated to identify all languages that are commonly used among South Asians. While this approach is likely to have good specificity for identifying South Asian home care clients, it will miss clients who would culturally identify as South Asian but who use other languages as their primary language [33]. It was not possible to differentiate cultural subpopulations of South Asian clients because of privacy concerns related to reidentification risk. The interRAI HC assessments data were anonymized by Ontario Health prior to transfer to the University of Waterloo. Use of these data was approved by the Office of Research Ethics at the University of Waterloo.

### 2.6 Bias

Reliability and validity of the interRAI HC assessment has been established previously. [28, 32, 34, 35, 36, 37, 38, 39, 40]. The completion of the interRAI HC is a mandatory requirement for all home care clients expected to be on service for at least 60 days. This excludes short stay home care clients who tend to receive on the interRAI CA, and that population has less complex care needs than long stay clients. Assessments are completed by trained health professionals with cultural competence being an expected part of the clinician's expertise, and translators are used at the time of assessment, if needed. interRAI assessments have been used internationally in research in over 30 countries indicating cross‐cultural applicability [31, 37, 39, 41, 42, 43, 44].

### 2.7 Sample Size

The study sample of 428,373 individuals included first assessments of all clients assessed in the study period making this a population level analysis.

### 2.8 Statistical Methods

Our analytic approach includes a sample description with comparisons of the distributional properties of depression diagnosis and CMS scores among South Asian and general home care clients. Chi‐square tests of significance were used to test bivariate associations. Multivariate logistic regression models were used to examine the risk factors for depression diagnosis based on demographic characteristics, known indicators of depression (e.g., pain, sleep disturbance, fatigue, loss of appetite), South Asian background, and other potential risk factors for depression. Our multivariate analyses included three main approaches. First, we examined the associations of South Asian status with a depression diagnosis while controlling for demographic factors (age, gender), the CMS score for depressive symptoms, and other clinical covariates that may be indicators associated with depression. We included only covariates significant at the *p* < 0.05 level. Next, we expanded this model to include additional variables that could be risk factors for depression. Finally, we checked for interaction terms between South Asian status and other covariates. We reported c statistics as a measure of model performance. We used SAS software version 9.4 for all analyses (SAS Institute Inc.)

## 3 Results

### 3.1 Participants

Table 1 summarizes the demographic and clinical characteristics of South Asian home care clients and all others representing the general population of home care recipients. All differences reported in this table are significant at the *p* < 0.0001 level. While the age distributions were similar for both groups, there was a higher percentage of persons 85 years or older in the general home care population than South Asian clients. In both cases, over 85% of clients were 65 or older. The gender distribution was different; however, the absolute differences were not substantively large (female 62.5% vs. 59.9%). However, South Asian clients were considerably more likely to be married (48.8% vs. 39.6%) than the general home care population. There were modest absolute differences in the severity of cognitive impairment in the two subpopulations, but these differences were again not substantively important.

**TABLE 1:** Sample characteristics by language group, ontario home care clients, 2018–2022.

Variable	South asians (*n* = 8618) In (%)	Others (*n* = 419,755) In (%)	Chi‐square and *p*‐value
Age group (in years)			
18–44	2.0	2.9	
45–64	9.1	12.0	416.6 *p* < 0.0001
65–74	18.8	17.0	
75–84	39.9	31.0	
85+	30.2	37.1	
Gender			
female	62.5	59.9	22.8 *p* < 0.0001
Male	37.5	40.1	
Marital status			
Married	48.8	39.6	299.4 *p* < 0.0001
Not married	51.2	60.4	
Cognitive performance scale			
0	17.5	22.5	
1–2	59.9	59.1	178.2 *p* < 0.0001
3+	22.7	18.4	
Depression diagnosis			
present	13.3	21.5	336.0 *p* < 0.0001
Not present	86.7	78.6	
Composite Mood scale score			
None	43.5	48.5	
Moderate	31.0	28.2	85.9 *p* < 0.0001
High	17.9	16.6	
severe	7.6	6.7	

### 3.2 Outcome Data

**FIGURE 1:**
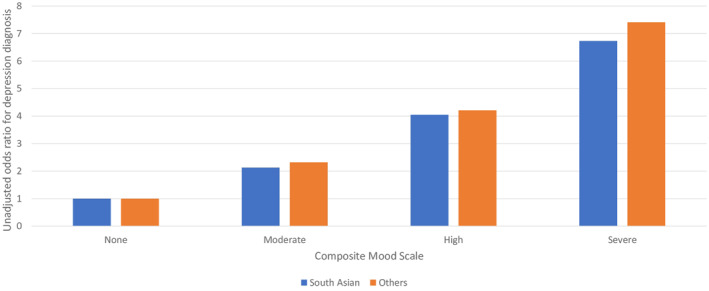
Unadjusted odds ratios for depression diagnosis by composite mood scale score and language group.

**FIGURE 2:**
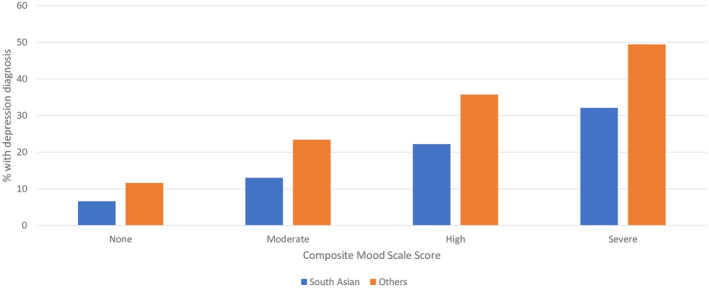
Percentage of home care clients with depression diagnosis by composite mood scale score.

The general home care population had a notably higher rate of depression diagnosis; however, the distributions of the CMS in the two subpopulations were similar. A somewhat large percentage of the general home care population had a CMS score of zero (48.5%) compared to South Asian home care clients (43.5%). Figure 1 shows the association between the CMS severity scores and the unadjusted odds of a depression diagnosis. In both subpopulations, there is a substantial and comparable increase in the *odds* of a depression diagnosis being present with higher scores on the CMS. However, Figure 2 shows that for every level of the CMS, the *percentage* of clients with a depression diagnosis is substantially lower among South Asian home care clients than the general population of home care recipients.

### 3.3 Main Results

Table 2 shows two multivariate models for the presence of a depression diagnosis. Model A considers demographic variables and clinical symptoms that are commonly reported to be associated with a depression diagnosis. For all home care clients, the odds of a depression diagnosis are lower among older age groups, and the highest odds of this diagnosis are among those under 65. Female home care clients have 1.46 times greater odds of a depression diagnosis. Any cognitive impairment is associated with increased odds of depression diagnosis. For every one‐point increment in the Pain scale, there is a 1.08 increased odds of depression diagnosis, which translates to an odds ratio of 1.36 for a maximum Pain scale score of 4. The effect of fatigue was less pronounced, with odds ratios of 1.13 and 1.06 for moderate and severe fatigue, respectively, and sleep disturbance was insignificant in this model. For every one‐point increase in the CMS score, the odds of a depression diagnosis were 1.24 times greater, which would translate to an odds ratio of 6.93 for the maximum score of 9. After adjusting for all of the abovementioned covariates, the odds of a depression diagnosis among South Asian client were 0.48 (95% CI 0.45–0.52) in Model A.

**TABLE 2:** Multivariate models for presence of depression diagnosis among ontario home care clients.

Variable	Model A		Model B	
	Parameter estimate (SE)	Odds ratio (95% CL)	Parameter estimate (SE)	Odds ratio (95% CL)
Age group in years (ref = 18–44)				
45–64	0.22 (0.02)	1.26 (1.20–1.32)	0.24 (0.02)	1.27 (1.21–1.33)
65–74	0.002 (0.02)	1.00 (0.96–1.05)	0.05 (0.02)	1.05 (1.00–1.10)
75–84	−0.39 (0.02)	0.68 (0.65–0.71)	−0.33 (0.02)	0.72 (0.69–0.75)
85+	−0.80 (0.02)	0.45 (0.43–0.47)	−0.76 (0.02)	0.47 (0.45–0.49)
Gender (ref = male)				
Female	0.38 (0.008)	1.46 (1.43–1.48)	0.34 (0.009)	1.40 (1.38–1.43)
Cognitive performance scale (ref = 0)				
1–2	0.45 (0.01)	1.57 (1.54–1.60)	0.46 (0.01)	1.58 (1.55–1.64)
3+	0.41 (0.01)	1.50 (1.46–1.54)	0.47 (0.01)	1.60 (1.55–1.64)
Pain scale (continuous)	0.08 (0.004)	1.08 (1.08–1.09)	0.08 (0.004)	1.09 (1.08–1.09)
Sleep problems (ref = not present)				
Present	0.006 (0.008)	1.01 (0.99–1.02)	0.008 (0.009)	1.01 (0.99–1.03)
Fatigue (ref = none)				
Moderate	0.12 (0.01)	1.13 (1.10–1.15)	0.15 (0.01)	1.16 (1.13–1.18)
Severe	0.06 (0.01)	1.06 (1.04–1.08)	0.12 (0.01)	1.13 (1.11–1.16)
Infrequent Meals (ref = not present)				
Present	−0.09 (0.01)	0.92 (0.89–0.94)	−0.03 (0.01)	0.97 (0.95–1.00)
Composite Mood scale score (continuous)	0.22 (0.001)	1.24 (1.24–1.25)	0.22 (0.002)	1.25 (1.24–1.25)
Language (ref = others)				
South Asian	−0.73 (0.03)	0.48 (0.45–0.52)	−0.69 (0.03)	0.50 (0.47–0.54)
Marital status (ref = not married)				
Married			−0.13 (0.01)	0.88 (0.87–0.90)
Live alone (ref = no)				
Yes			0.07 (0.01)	1.07 (1.05–1.09)
Major stressors (ref = no)				
Yes			−0.07 (0.009)	0.93 (0.92–0.95)
ADL Hierarchy scale (ref = 0)				
1–2			−0.008 (0.01)	0.99 (0.97–1.01)
3+			−0.07 (0.01)	0.94 (0.92–0.96)
CHESS scale (ref = 0)				
1–2			−0.05 (0.01)	0.95 (0.93–0.97)
3+			−0.18 (0.01)	0.84 (0.82–0.86)
Strong and supportive family (ref = no)				
Yes			−0.18 (0.01)	0.94 (0.82–0.86)
Economic trade offs (ref = no)				
yes			0.20 (0.02)	1.23 (1.18–1.28)
c	0.712		0.714	

Model B expands the multivariate model to include additional potential protective and risk factors in the model. The odds of a depression diagnosis were *lower* for persons who were married, had moderate or worse ADL impairment, had unstable health, experienced recent stressful events, or had strong and supportive family members. The odds of the diagnosis were *greater* for clients aged between 45 and 64 years, females, who lived alone or who were making economic trade‐offs in purchasing food, shelter, clothing, or medications, and who have moderate to severe fatigue. After adjusting for all of these additional covariates, the odds of depression diagnosis were 0.50 (95% CI 0.47–0.54) for South Asian home care clients. For both models, the c statistic was approximately 0.71.

## 4 Discussion

Our study findings show that the demographic and cognitive characteristics of South Asian home care clients are similar to the general home care population. While about 66% of South Asian clients have moderate or worse indicators of mood disturbance compared with 61% of the general home care population, the percentage with existing depression diagnoses is about half in the South Asian subpopulation compared with other home care clients. We also showed that the relationship between mood disturbance and depression diagnosis was very similar between the two groups; however, for every level of mood disturbance, the percentage of clients with an existing depression diagnosis was much lower among South Asians.

Importantly, multivariate analyses showed that this disparity could not be attributed to differences in demographic factors, physical comorbidities, protective or risk factors, or depressive symptoms that might be associated with clinical diagnosis. Thus, the under‐diagnosis is unlikely to be due to clinical profile differences and more plausibly reflects systemic or cultural barriers in the recognition process.

One factor that may explain these findings is the influence of cultural norms around emotional expression and mental illness among South Asians. Stigma around mental health remains pervasive in many South Asian cultures. Clients and caregivers may actively avoid discussing emotional distress due to fears of shame and social exclusion, this cultural hesitation can suppress reporting even when mood disturbance is present and clinically observable, creating a gap between symptom burden and diagnosis. Our study's results are consistent with this interpretation, as mood disturbance captured by the interRAI HC was evident, but formal diagnosis lagged significantly. Another explanation may lie in clinician‐patient communication gaps. South Asian clients may experience language barriers or difficulty articulating distress in ways that are clinically recognized, while clinicians may lack the cultural competence to probe or interpret these concerns effectively.

The strong association between CMS scores and depression diagnosis highlights the potential of standardized tools like the interRAI HC to effectively detect mood disturbances in home care settings. However, the impact of these assessments relies heavily on how well the findings are communicated to and utilized by primary care providers. Thus, to fully realize the benefits of systematic assessment, it is essential to ensure that interRAI HC results are meaningfully integrated into care planning and decision‐making processes within the broader healthcare team.

Home care within South Asian communities is influenced by unique cultural, social, and familial dynamics, which can affect both accesses to care and symptom disclosure. Recognizing these dynamics is essential for fostering a holistic and inclusive model of care for South Asian clients receiving home services. Understanding the cultural nuances of depression within South Asian communities is crucial for designing effective interventions. Tiwari and Wang [45] highlighted the impact of cultural diversity on mental health service utilization patterns, while Naeem et al. [46] proposed culturally adapted interventions aligning with cultural values. However, ethnic minority groups, including South Asians, are often underserved within healthcare systems and lack adequate community support services, as observed by [47, 48]. Further, research by Islam et al. [21] uncovers the challenges faced by South Asian youth in accessing mental health services in Canada, emphasizing the need for culturally tailored support systems. Grace et al. [49] emphasized the mental health disparities experienced by ethnocultural minorities, emphasizing the importance of culturally sensitive interventions.

In the absence of a standardized assessment like the interRAI HC, South Asian clients with depression may not report mood disturbances on their own and they may not present with characteristic features to healthcare providers. Further evaluation may reveal more marked features and provide indications of clinical significance. Another explanation may be stigma. Despite high percentages of clients with mood disturbance, there exists a significant gap in the formal recognition of depression within this population, potentially attributed to cultural stigma [50]. There are important cross‐cultural variations in the degree of stigma associated with mental health diagnoses [51]. It may be the case that clinicians are reluctant to formalize a depression diagnosis among South Asian clients because of concerns about stigma. On the other hand, it may also be the case that South Asian clients and their caregivers under‐report that they have had a depression diagnosis because of that stigma. These results clearly demonstrate the value of standardized clinical assessment of home care clients' physical and mental health needs using the interRAI HC. If one relied on diagnostic information alone, there would likely be a substantial under‐detection and failure to respond to conditions like mood disturbance.

These results have important implications for clinical practice, policy, and research. They show that mood disturbances are just as common among South Asian home care clients as they are in the general home care population, yet they are frequently under‐recognized by health professionals. This underscores the necessity for an integrated continuum of mental health care in home care settings. Integration involves a comprehensive approach, combining social and health services to address seniors' needs through aligned financial and administrative incentives, standardized and thorough assessments, and support from interprofessional clinical teams [52]. Home care and primary care professionals must collaborate in a unified approach to ensure that all relevant health professionals are aware of the significant physical and mental health needs identified through standardized clinical assessments, thus providing a seamless continuum of mental health care. From a policy perspective, efforts should be undertaken to increase community awareness, reduce stigma associated with mental health concerns, improve the integration of mental health care into primary health care, involve multidisciplinary teams and community organizations, and increase access to culturally appropriate mental health services for South Asian clients. In addition, the present findings provide evidence for the validity of the CMS as a measure of mood disturbance among South Asian clients, given its comparable performance about depression diagnosis as found in the general population and other studies [26].

There are several next steps to pursue in research. First, it would be useful to conduct similar research in South Asian nations to determine whether these results are unique to Canada. Second, it would be useful to conduct intervention studies to determine what interventions might be cost‐effective in South Asian populations compared with other home care clients.

## 5 Limitations

The limited precision of identifying cultural background based on primary language is a notable limitation. This may under‐detect fluently multilingual South Asians and it also represents only one dimension of culture. In addition, it would have been interesting to identify other cultural strata based on recency of immigration or place of birth; however, these were not available because they were not collected, or they were suppressed for privacy reasons. In addition, medication use was not considered here because all drug data were captured in free text form without standardized ATC codes. Finally, it would be helpful to conduct additional validation studies of the CMS in South Asian populations. Several interRAI studies are currently underway in India using a self‐reported version of the scale, but these results have not yet been published.

## 6 Conclusion

In conclusion, our study highlights a concerning under‐recognition and under‐diagnosis of depression among South Asian home care clients compared to the general home care population, despite similar demographic and cognitive characteristics. This discrepancy suggests potential barriers to accurate diagnosis, including cultural competence among health care providers and stigma surrounding mental health issues within South Asian communities. Addressing these challenges is imperative to ensure appropriate care and support for South Asian individuals experiencing mood disturbances. Our findings underscore the importance of standardized clinical assessment instruments such as the interRAI HC in identifying mental health needs among home care clients, emphasizing the need for collaboration between home care and primary care providers to deliver comprehensive care. Policy efforts should focus on increasing awareness, reducing stigma, and enhancing access to culturally appropriate mental health services for South Asian clients. Moving forward, research should explore these issues in diverse settings and evaluate the effectiveness of interventions tailored to South Asian populations to promote mental well‐being within these communities.

## Funding

The authors have nothing to report.

## Ethics Statement

Access to these deidentified data was possible through a data sharing agreement between University of Waterloo and Ontario Health. Ethics clearance for secondary analysis of these data was obtained from the Office of Research Ethics (ORE#30173). Assessments are done as part of routine care and are covered by organizational consent to treatment.

## Consent

The authors have nothing to report.

## Conflicts of Interest

The authors declare no conflicts of interest.

## Data Availability

The data used in this research can be accessed via an application for research use to the Canadian Institute for Health Information.

## References

[1] Global Health Data Exchange , Depressive Disorders – Level 3 Cause (Institute for Health Metrics and Evaluation, 2019), https://ghdx.healthdata.org.

[2] World Health Organization , Depressive Disorder (Depression), 2023): [Fact sheet], https://www.who.int/news‐room/fact‐sheets/detail/depression.

[3] World Health Organization , “World Mental Health Report: Transforming Mental Health for all,”2022, https://www.who.int/publications/i/item/9789240049338.

[4] D. G. Blazer , C. F. Hybels , and C. F. Pieper , “The Association of Depression and Mortality in Elderly Persons: A Case for Multiple, Independent Pathways,” Journals of Gerontology. Series A, Biological Sciences and Medical Sciences 56, no. 8 (2001): M505–M509, 10.1093/gerona/56.8.m505. 11487603

[5] K. Moss , F. Scogin , E. Di Napoli , and A. Presnell , “A Self‐Help Behavioral Activation Treatment for Geriatric Depressive Symptoms,” Aging & Mental Health 16, no. 5 (2012): 625–635, 10.1080/13607863.2011.651435. 22304676

[6] Y. Zenebe , B. Akele , M. W/Selassie , and M. Necho , “Prevalence and Determinants of Depression Among Old Age: A Systematic Review and Meta‐Analysis,” Annals of General Psychiatry 20, no. 1 (2021): 55, 10.1186/s12991-021-00375-x. 34922595 PMC8684627

[7] D. W. Lai and S. Surood , “Socio‐Cultural Variations in Depressive Symptoms of Ageing South Asian Canadians,” Asian Journal of Gerontology & Geriatrics 3, no. 2 (2008): 84–91, http://hdl.handle.net/1880/49368.

[8] D. W. Lai and S. Surood , “Predictors of Depression in Aging South Asian Canadians,” Journal of cross‐cultural gerontology 23, no. 1 (2008): 57–75, 10.1007/s10823-007-9051-5. 17990088

[9] A. Karasz , K. Dempsey , and R. Fallek , “Cultural Differences in the Experience of Everyday Symptoms: A Comparative Study of South Asian and European American Women,” Culture, Medicine and Psychiatry 31, no. 4 (2007): 473–497, 10.1007/s11013-007-9066-y. 17985219

[10] A. G. Ryder , J. Yang , and S. J. Heine , “Somatization vs. Psychologization of Emotional Distress: a Paradigmatic Example for Cultural Psychopathology,” in Online Readings in Psychology and Culture, W. J. Lonner , D. L. Dinnel , S. A. Hayes , and D. N. Sattler , eds. 9th ed. (Center for Cross‐Cultural Research, Western Washington University, 2002).

[11] B. B. Sethi , “Epidemiology of Depression in India,” supplement, Psychopathology 19, no. Suppl 2 (1986): 26–36, 10.1159/000285129. 3554307

[12] V. Patel and M. Prince , “Global Mental Health: A New Global Health Field Comes of Age,” JAMA 303, no. 19 (2010): 1976–1977, 10.1001/jama.2010.616. 20483977 PMC3432444

[13] A. Karasz , F. Gany , J. Escobar , et al., “Mental Health and Stress Among South Asians,” supplement, Journal of Immigrant and Minority Health 21, no. Suppl 1 (2019): 7–14, 10.1007/s10903-016-0501-4. 27848078 PMC5643212

[14] F. Islam , N. Khanlou , and H. Tamim , “South Asian Populations in Canada: Migration and Mental Health,” BMC Psychiatry 14, no. 1 (2014): 154, 10.1186/1471-244X-14-154. 24884792 PMC4052282

[15] K. Singh , “Suicide Among Immigrants to Canada From the Indian Subcontinent,” Canadian journal of psychiatry/Revue canadienne de psychiatrie 47, no. 5 (2002): 487, 10.1177/070674370204700519. 12085689

[16] L. J. Kirmayer , M. Weinfeld , G. Burgos , G. G. du Fort , J. C. Lasry , and A. Young , “Use of Health Care Services for Psychological Distress by Immigrants in an Urban Multicultural Milieu,” Canadian journal of psychiatry. Revue canadienne de psychiatrie 52, no. 5 (2007): 295–304, 10.1177/070674370705200504. 17542380

[17] T. M. Gadalla , “Ethnicity and Seeking Treatment for Depression: A Canadian National Study,” Canadian Ethnic Studies 41, no. 3 (2010): 233–245, 10.1353/ces.2010.0042.

[18] A. Durbin , R. Moineddin , E. Lin , L. S. Steele , and R. H. Glazier , “Mental Health Service Use by Recent Immigrants From Different World Regions and by Non‐Immigrants in Ontario, Canada: A Cross‐Sectional Study,” BMC Health Services Research 15, no. 1 (2015): 336, 10.1186/s12913-015-0995-9. 26290068 PMC4546085

[19] Y. K. Dwivedi , E. Ismagilova , D. L. Hughes , et al., “Setting the Future of Digital and Social Media Marketing Research: Perspectives and Research Propositions,” International Journal of Information Management 59 (2021): 102168, 10.1016/j.ijinfomgt.2020.102168.

[20] S. Guruge and N. Khanlou , “Intersectionalities of Influence: Researching the Health of Immigrant and Refugee Women,” Canadian Journal of Nursing Research 36, no. 3 (2004): 32–47, https://pubmed.ncbi.nlm.nih.gov/15551661/. 15551661

[21] F. Islam , A. Multani , M. Hynie , Y. Shakya , and K. McKenzie , “Mental Health of South Asian Youth in Peel Region, Toronto, Canada: A Qualitative Study of Determinants, Coping Strategies and Service Access,” BMJ Open 7, no. 11 (2017): e018265, 10.1136/bmjopen-2017-018265. PMC5695456 29101148

[22] J. Edwards , M. Hu , A. Thind , S. Stranges , M. Chiu , and K. K. Anderson , “Gaps in Understanding of the Epidemiology of Mood and Anxiety Disorders Among Migrant Groups in Canada: A Systematic Review,” Canadian Journal of Psychiatry/Revue Canadienne de Psychiatrie 64, no. 9 (2019): 595–606, 10.1177/0706743719839313. 31129987 PMC6699028

[23] R. Mooney , D. Trivedi , and S. Sharma , “How do People of South Asian Origin Understand and Experience Depression? A Protocol for a Systematic Review of Qualitative Literature,” BMJ Open 6, no. 8 (2016): e011697, 10.1136/bmjopen-2016-011697. PMC5013340 27577586

[24] Statistics Canada , “Portrait of the South Asian Populations in Canada: Diversity and Socioeconomic Outcomes (Catalogue No. 89‐657‐X),”2025, https://www150.statcan.gc.ca/n1/en/catalogue/89‐657‐x.

[25] D. Chowdhury , C. Tong , K. Lopez , E. Neiterman , and P. Stolee , “When in Rome…”: Structural Determinants Impacting Healthcare Access, Health Outcomes, and Well‐Being of South Asian Older Adults in Ontario Using a Multilingual Qualitative Approach,” Frontiers in Public Health 12 (2024): 1405851: Article 1405851, 10.3389/fpubh.2024.1405851. 39741940 PMC11685128

[26] J. P. Hirdes , J. N. Morris , C. M. Perlman , et al., “Mood Disturbances Across the Continuum of Care Based on Self‐Report and Clinician Rated Measures in the interRAI Suite of Assessment Instruments,” Frontiers in Psychiatry 13 (2022): 787463, 10.3389/fpsyt.2022.787463. 35586405 PMC9108209

[27] J. N. Morris , I. Carpenter , K. Berg , and R. N. Jones , “Outcome Measures for Use With Home Care Clients,” supplement, Canadian Journal on Aging/La Revue canadienne du vieillissement 19, no. S2 (2000): 87–105, 10.1017/S0714980800014747.

[28] F. Landi , E. Tua , G. Onder , et al., “Minimum Data Set for Home Care: A Valid Instrument to Assess Frail Older People Living in the Community,” Medical Care 38, no. 12 (2000): 1184–1190, 10.1097/00005650-200012000-00005. 11186297

[29] J. P. Hirdes , D. H. Frijters , and G. F. Teare , “The MDS‐CHESS Scale: A New Measure to Predict Mortality in Institutionalized Older People,” Journal of the American Geriatrics Society 51, no. 1 (2003): 96–100, 10.1034/j.1601-5215.2002.51017.x. 12534853

[30] C. J. Sinn , J. P. Hirdes , J. W. Poss , V. M. Boscart , and G. A. Heckman , “Implementation Evaluation of a Stepped Approach to Home Care Assessment Using interRAI Systems in Ontario, Canada,” Health and Social Care in the Community 30, no. 6 (2022): 2341–2352, 10.1111/hsc.13784. 35484905 PMC10078667

[31] I. Carpenter and J. P. Hirdes , “Using interRAI Assessment Systems to Measure and Maintain Quality of long‐term Care,” in A Good Life in Old Age? Monitoring and Improving Quality of long‐term Care (OECD Publishing, 2013), 93–139: OECD/European Commission, 10.1787/9789264194564-en.

[32] J. P. Hirdes , G. Ljunggren , J. N. Morris , et al., “Reliability of the interRAI Suite of Assessment Instruments: A 12‐Country Study of an Integrated Health Information System,” BMC Health Services Research 8, no. 1 (2008): 277, 10.1186/1472-6963-8-277. 19115991 PMC2631461

[33] R. Batista , A. T. Hsu , L. Bouchard , et al., “Ascertaining the Francophone Population in Ontario: Validating the Language Variable in Health Data,” BMC Medical Research Methodology 24, no. 1 (2024): 98, 10.1186/s12874-024-02220-7. 38678174 PMC11055282

[34] J. N. Morris , B. E. Fries , K. Steel , et al., “Comprehensive Clinical Assessment in Community Setting: Applicability of the MDS‐HC,” Journal of the American Geriatrics Society 45, no. 8 (1997): 1017–1024, 10.1111/j.1532-5415.1997.tb02975.x. 9256857

[35] A. D. Foebel , J. P. Hirdes , G. A. Heckman , M. J. Kergoat , S. Patten , and R. A. Marrie , and ideas PNC research team , “Diagnostic Data for Neurological Conditions in interRAI Assessments in Home Care, Nursing Home and Mental Health Care Settings: A Validity Study,” BMC Health Services Research 13, no. 1 (2013): 457, 10.1186/1472-6963-13-457. 24176093 PMC3893477

[36] J. P. Hirdes , J. W. Poss , L. Mitchell , L. Korngut , and G. Heckman , “Use of the interRAI CHESS Scale to Predict Mortality Among Persons With Neurological Conditions in Three Care Settings,” PLoS One 9, no. 6 (2014): e99066, 10.1371/journal.pone.0099066. 24914546 PMC4051671

[37] H. Kim , Y. I. Jung , M. Sung , J. Y. Lee , J. Y. Yoon , and J. L. Yoon , “Reliability of the interRAI Long Term Care Facilities (LTCF) and interRAI Home Care (HC),” Geriatrics and Gerontology International 15, no. 2 (2015): 220–228, 10.1111/ggi.12330. 25163513

[38] J. De Almeida Mello , K. Hermans , C. Van Audenhove , J. Macq , and A. Declercq , “Evaluations of Home Care Interventions for Frail Older Persons Using the interRAI Home Care Instrument: A Systematic Review of the Literature,” Journal of the American Medical Directors Association 16, no. 2 (2015): 173.e1–173.e10, 10.1016/j.jamda.2014.11.007. 25512214

[39] S. E. Hogeveen , J. Chen , and J. P. Hirdes , “Evaluation of Data Quality of interRAI Assessments in Home and Community Care,” BMC Medical Informatics and Decision Making 17, no. 1 (2017): 150, 10.1186/s12911-017-0547-9. 29084534 PMC5663080

[40] M. Ulate , A. Molinari , A. Mahmoudi Asl , et al., “Comprehensive Geriatric Assessments for Long‐Term and Home Care Settings and Digital Platforms Available to Support their Applicability: A Systematic Review,” supplement, Alzheimer's & Dementia 17, no. S10 (2021): e057529, 10.1002/alz.057529.

[41] I. Carpenter , G. Gambassi , E. Topinkova , et al., “Community Care in Europe. the Aged in Home Care Project (AdHOC),” Aging Clinical and Experimental Research 16, no. 4 (2004): 259–269, 10.1007/BF03324550. 15575119

[42] L. C. Gray , N. M. Peel , A. P. Costa , et al,. “Profiles of Older Patients in the Emergency Department: Findings From the interRAI Multinational Emergency Department Study,” Annals of Emergency Medicine 62, no. 5 (2013): 467–474, 10.1016/j.annemergmed.2013.05.008. 23809229

[43] J. Hikaka , R. Abey‐Nesbit , Z. Wu , et al., “Changes in Indicators of Well‐Being on Moving From Home to long‐term Care for Māori in Aotearoa New Zealand: A Retrospective Cohort Study,” Australasian Journal on Ageing 43, no. 4 (2024): 818–827, 10.1111/ajag.13361. 39135395 PMC11671709

[44] J. F. Hikaka , A. H. Y. Chan , B. Meehan , et al., “Using interRAI Assessment for Research: Developing a National Research Agenda in Aotearoa New Zealand,” Journal of the American Medical Directors Association 25, no. 6 (2024): 104998, 10.1016/j.jamda.2024.03.109. 38643969

[45] S. K. Tiwari and J. Wang , “Ethnic Differences in Mental Health Service Use Among White, Chinese, South Asian and South East Asian Populations Living in Canada,” Social Psychiatry and Psychiatric Epidemiology 43, no. 11 (2008): 866–871, 10.1007/s00127-008-0373-6. 18500481

[46] F. Naeem , A. Tuck , B. Mutta , et al., “Protocol for a Multi‐Phase, Mixed Methods Study to Develop and Evaluate Culturally Adapted CBT to Improve Community Mental Health Seravices for Canadians of South Asian Origin,” Trials 22, no. 1 (2021): 600, 10.1186/s13063-021-05547-4. 34488853 PMC8419942

[47] B. W. Chang and J. P. Hirdes , “A cross‐sectional Study to Compare Caregiver Distress Among Korean Canadian, Chinese Canadian, and Other Canadian Home Care Clients,” Sage Open 5, no. 2 (2015): 2158244015591824, 10.1177/2158244015591824.

[48] A. S. Derr , “Mental Health Service Use Among Immigrants in the United States: A Systematic Review,” Psychiatric Services 67, no. 3 (2016): 265–274, 10.1176/appi.ps.201500004. 26695493 PMC5122453

[49] S. L. Grace , Y. Tan , R. A. Cribbie , H. Nguyen , P. Ritvo , and J. Irvine , “The Mental Health Status of Ethnocultural Minorities in Ontario and Their Mental Health Care,” BMC Psychiatry 16, no. 1 (2016): 47, 10.1186/s12888-016-0759-z. 26915910 PMC4768406

[50] R. Gater , B. Tomenson , C. Percival , et al., “Persistent Depressive Disorders and Social Stress in People of Pakistani Origin and White Europeans in UK,” Social Psychiatry and Psychiatric Epidemiology 44, no. 3 (2009): 198–207, 10.1007/s00127-008-0426-x. 18726242

[51] H. U. Ahmed , M. D. Hossain , A. Aftab , et al., “Suicide and Depression in the World Health Organization South‐East Asia Region: A Systematic Review,” WHO South‐East Asia journal of public health 6, no. 1 (2017): 60–66, 10.4103/2224-3151.206167. 28597861

[52] A. Moore , C. Patterson , J. White , et al., “Interprofessional and Integrated Care of the Elderly in a Family Health Team,” Canadian family physician/Medecin de famille canadien 58, no. 8 (2012): e436–e441, https://pubmed.ncbi.nlm.nih.gov/22893345/. 22893345 PMC3419000

